# Antioxidative effects of supplementing linseed oil-enriched diets with α-tocopherol, ascorbic acid, selenium, or their combination on carcass and meat quality in broilers

**DOI:** 10.3382/ps/pez389

**Published:** 2019-07-15

**Authors:** J Leskovec, A Levart, L Perić, M Đukić Stojčić, V Tomović, T Pirman, J Salobir, V Rezar

**Affiliations:** 1 Biotechnical Faculty, Department of Animal Science, University of Ljubljana, 1000 Ljubljana, Slovenia; 2 Faculty of Agriculture, Department of Animal Science, University of Novi Sad, 21000 Novi Sad, Serbia; 3 Faculty of Technology Novi Sad, University of Novi Sad, 21000 Novi Sad, Serbia

**Keywords:** vitamin E, vitamin C, selenium, broiler, meat quality

## Abstract

In a previous study, we examined the synergistic effects of the dietary supranutritional supplementation with vitamin E, vitamin C, and Se on the in vivo antioxidative status of broilers under conditions of dietary oxidative stress induced by feeding a diet high in n-3 PUFA. In this study, we examined the effect of their inclusion on the quality characteristics and oxidative stability of raw or cooked meat, both fresh or after a long-term frozen storage. Four hundred 21-day-old Ross 308 male broilers were allocated to 5 experimental groups fed 5% linseed oil-enriched finisher diets (days 21 to 40): Cont (recommended levels of vitamin E, C, and selenium), +E (200 IU vitamin E/kg feed), +C (250 mg vitamin C/kg feed), +Se (0.2 mg selenium/kg feed), or +ECSe (concentrations as in the sole supplementation, combined). Animal performance and carcass characteristics were monitored at the age of 40 D. Breast meat samples of 12 chickens per group were analysed fresh, fresh after frozen storage, cooked fresh, and cooked after frozen storage (2 × 2 factorial design) for parameters of meat quality (water-holding capacity—WHC, pH, and color) and oxidative stability (concentrations of vitamin E, malondialdehyde—MDA, antioxidant capacity of the water-soluble compounds—ACW, and fatty acid composition).

Vitamin E alone (+E) and combined with Se and vitamin C (+ECSe) increased the α-tocopherol concentration in breast muscle, and showed similar protective effects against lipid peroxidation measured as MDA regardless of the frozen storage or cooking. The sole supplementation of vitamin C or selenium showed no effects on the meat quality parameters. In conclusion, the dietary supranutritional inclusion of vitamin E inhibited the lipid peroxidation in fresh, frozen stored, cooked fresh, and frozen stored meat in broilers fed with diets rich in n-3 PUFAs. Even though no clear synergistic effects of the supranutritional supplementation of vitamin C and Se with vitamin E were detected, their dietary inclusion did not negatively affect broilers carcass and meat quality parameters.

## INTRODUCTION

Recent human nutrition recommendations favor the consumption of n-3 polyunsaturated fatty acids (**PUFA**), since they possess numerous positive properties for the human health. Therefore, many functional foods such as n-3 PUFA-enriched eggs and poultry meat and meat products were developed via inclusion of dietary oils rich in n-3 PUFA. On the other hand, changes towards an increased n-3 PUFA content in the fatty acid profile of meat can raise the oxidative stress in poultry (Juskiewicz et al., 2017; Voljč et al., 2011). Lipid oxidation and discoloration are the major causes of meat quality deterioration during storage (Ryu et al., 2005, 2006). Therefore, the inclusion of dietary n-3 PUFA increases the requirements for antioxidants and particularly for vitamin E, the most important lipid-soluble antioxidant (Jensen et al., 1998; Raederstorff et al., 2015).

Vitamin E can reduce the extent of lipid oxidation in meat and meat products, diminish changes in color, flavor, texture, drip loss, and consequently improve their oxidative stability (Cheng et al., 2016; Jensen et al., 1998). The dietary supplementation with vitamin E has positive effects on meat derived from chickens reared under heat stress, as it lowers the lipid peroxidation measured as the level of malondialdehyde (**MDA**) in breast meat (Habibian et al., 2016). It also provides protection against the oxidative stress induced by the augmented dietary inclusion of n-3 PUFA in broilers, indicated as an increase of α-tocopherol in plasma and meat, a decrease of MDA in plasma, and protection of DNA against oxidative damage (Tomažin et al., 2013).

Besides vitamin E, vitamin C is also an important hydrophilic antioxidant. Due to the fact that vitamin C is endogenously synthesized by the liver in poultry, no dietary inclusion is necessary according to the commercial nutrition recommendations for broilers (Aviagen, 2014). Furthermore, vitamin C can effectively restore the activity of vitamin E (Forman et al., 2014). Previous studies reported that dietary vitamin C alleviated stress induced by heat (Sahin et al., 2003), copper (Cinar et al., 2014), and dexamethasone (El-Senousey et al., 2018) in poultry. According to the existing literature, dietary vitamin C affects oxidative stress and possibly meat quality in poultry, although some trials concluded that dietary vitamin C did not have any effect on color, odor, flavor, and overall acceptability of breast and thigh meat (Skrivan et al., 2012).

Selenium is part of many selenoproteins with antioxidative activity (Surai, 2002a). Selenium-dependent enzymes could have synergistic effects when combined with vitamins E and C, which is theoretically known. As reported, dietary Se improved meat quality alone or in combination with other antioxidants (Surai, 2002b), but it has never been assessed together with vitamins E and C. Also, the dietary supplementation with Se alleviated the oxidative stress in poultry induced by heat stress (Habibian et al., 2016) and dietary Se deficiency (Halliwell and Gutteridge, 1999), but not when induced by high n-3 PUFA dietary levels (Malayoglu et al. 2009). Dietary Se increased the accumulation of antioxidants in liver and plasma, and improved the immune response (Karadas et al., 2016). Moreover, Se deficiency caused similar effects as that of vitamin E deficiency, especially regarding lipid peroxidation (Halliwell and Gutteridge, 1999).

The antioxidant network is very complex and some antioxidants work synergistically. The meat quality is negatively affected by oxidative stress and therefore the inclusion of different antioxidants with synergistic effects could have positive effects. It is well known that vitamin C reduces vitamin E radical to active form (Chan, 1993), and that Se-dependent thioredoxin reductase regenerates vitamin C (May et al., 1998). Furthermore, Se-dependent glutathione peroxidase reduces lipid peroxides (Beck, 2007) and therefore supports the activity of vitamin E. Some trials in poultry observed synergistic activities: vitamin E and Se (Habibian et al., 2016; Harsini et al., 2012) and vitamin C and Se (Skrivan et al., 2012) in broilers, vitamins E and C in broiler breeder hens under heat stress (Jena et al., 2013), and vitamin E and Se in quails under normal rearing conditions (Zancanela et al., 2018). The combination of the 3 antioxidants was assessed only in fish, in which synergistic effects on the oxidative stress were reported (Park et al., 2016). On the other hand, in our previously published study, synergistic effects of vitamins E and C and Se on in vivo indicators of oxidative stress were not observed (Leskovec et al., 2018).

To the best of our knowledge, no trials have assessed the effects of the combined dietary supranutritional inclusion of vitamin E, vitamin C, and Se on the carcass and meat quality characteristics of broilers. Therefore, the present study was aimed at determining whether, and if so to what extent, the supplementation with a combination of vitamins E, C, and selenium was superior to the sole supplementation of these antioxidants in broilers fed a n-3 PUFA-enriched diet. This was assessed on carcass traits and meat quality characteristics of fresh and frozen stored meat, either raw or cooked. Linseed oil was included at a level commonly used in practice in order to produce n-3 PUFA-enriched poultry meat and meat products.

## MATERIALS AND METHODS

In the present manuscript, a limited description of the materials and methods is given. For more detailed information, see Leskovec et al. (2018).

### Experimental Birds and Treatments

The trial was performed in 400 one-day-old male Ross 308 broiler chickens allocated into 5 groups with a complete block design. Chickens were fed a commercial starter diet during the first 12 D, a commercial grower diet up to day 20 and an experimental finisher diet until day 40. Feeds were formulated following the commercial nutrition specifications for Ross 308 broilers regarding vitamin E, vitamin C, and selenium (Aviagen, 2014), except the finisher diet. Oxidative stress was induced by the supplementation of 5% of linseed oil in the finisher diet, containing 50 IU vitamin E/kg and 0.3 mg Se/kg. The experimental diets were further supplemented with: no additives (Cont), 200 IU vitamin E/kg (+E), 250 mg vitamin C/kg (+C), 0.2 mg Se/kg (+Se), or a combination of the three (200 IU vitamin E/kg, 250 mg vitamin C/kg and 0.2 mg Se/kg; +ECSe) (Table 1). The temperature and lightning were maintained as suggested by Ross 308 recommendations. All the procedures were conducted according to the ethical norms of the EU Convention for the Protection of Vertebrate animals used for experimental and other scientific purposes, confirmed by Serbian authorities.

**Table 1.** Proximate composition and contents of Se, vitamin C, and α-tocopherol of the feed mixtures.

	Diets				
Constituents (g/kg)	Cont	+E	+C	+Se	+ECSe
Dry matter	877.7	879.7	879.1	879.9	878.5
Crude protein	183.9	186.0	188.1	183.2	189.7
Crude fat	59.8	58.2	59.6	62.4	58.9
Crude fibre	37.4	37.5	35.2	38.1	41.6
Crude ash	48.2	47.2	47.1	47.2	47.2
Nitrogen free extract	548.3	550.8	549.0	549.0	541.2
Se (mg/kg)	0.33	0.30	0.23	0.49	0.50
Vitamin C (mg/kg)	N.A.	N.A.	132.4	N.A.	129.4
α-tocopherol (mg/kg)	51.2	277.5	55.2	45.9	241.6

N.A. = not analysed.

Cont (commercially recommended levels of vitamin E, C and selenium), +E (Cont + 200 IU vitamin E/kg feed), +C (Cont + 250 mg vitamin C/kg feed), +Se (Cont + 0.2 mg selenium/kg feed), or +ECSe (Cont + 200 IU vitamin E, 250 mg vitamin C, 0.2 mg selenium/kg feed).

### Experimental Procedure and Sampling

Broilers were fed ad libitum and their performance was recorded weekly. At the age of 40 D, 12 broilers per group were slaughtered, and samples of breast muscle were collected.

### Carcass Characteristics

Slaughtering, dressing percentage evaluation, and carcass dissection were performed as described in Bogosavljevic-Boskovic et al. (2011).

### Meat Quality

The left side of the breast muscle was stored at 4°C, the analysis of physical parameters of meat quality were carried out 24 h post-mortem (water-holding capacity—**WHC**, pH, and color), 48 h post-mortem (pH and color), and 72 and 132 h post-mortem (color). Analyses of WHC were performed as described in Tomović et al. (2014). The pH value was measured using a portable pH meter (Consort T651, Turnhout, Belgium) equipped with and insertion electrode (Mettler Toledo, Greifensee, Switzerland) as in Tomović et al. (2016a). The meat color was measured using a Konica Minolta Chroma Meter CR-400 (Minolta Co., Ltd, Osaka, Japan) as in Tomović et al. (2016b).

To measure the influence of the heat treatment and frozen storage, the right half of the breast muscle was exposed to different treatments according to the process: fresh (not treated), cooked fresh (fresh meat heat-processed at 85°C for 60 min), frozen stored (frozen for 3 mo at −20°C), and cooked frozen stored (frozen for 3 mo at −20°C and heat-processed at 85°C for 60 min). Stored meat samples were kept in Ziploc polypropylene bags at −20°C. Cooking was performed in plastic tubes submerged in water (water bath LCS 0081 Lauda, Bartelt GmbH, Graz, Austria) for 60 min at 85°C. Prior to the analyses, samples were homogenized using liquid nitrogen and a knife mill (Grindomix GM200, Retsch GmbH and Co., Haan, Germany), and stored immediately (fresh) or after frozen storage (3 mo at −20°C) at −80°C.

The extent of the lipid oxidation and antioxidative properties of the breast muscles were monitored by measuring the fatty acid composition as in Park and Goins (1994), the MDA and vitamin E (α- and γ-tocopherol) levels as in Voljč et al. (2011), and the antioxidant capacity of the water-soluble compounds (**ACW**) using the photochemiluminiscence method following the protocol provided by the manufacturer (Photochem, Analytik Jena, Jena, Germany).

### Statistical Analyses

Data were statistically evaluated by the Mixed procedure of SAS software (Ver. 9.4; SAS Institute Inc. Cary, NC), as described in Leskovec et al. (2018). In the statistical model, the experimental group was included as a fixed effect and the replication pen as a random effect. For the repeated measures, a model with storage time as the fixed effect was used. Differences were considered significant at *P* < 0.05.

## RESULTS

The dietary supplements did not affect the dressing percentage and the carcass yield of breast, drumstick, wings, back, and abdominal fat (Table 2). Similarly, no effects on WHC, pH_24h_, pH_48h_ (Table 3), and meat color (L^*^, a^*^, b^*^) (Table 4) of breast muscle were observed. The frozen storage of breast meat caused a decrease in L^*^ and b^*^ values, but a^*^ was not influenced, regardless of the experimental group (Table 4).

**Table 2.** Effect of the dietary supplements on the dressing percentage (ready to grill), and carcass yields (%) of breasts, legs, wings, back, and abdominal fat.

	Diets						
	Cont	+E	+C	+Se	+ECSe	SEM	*P*-value
Dressing percentage (%)	69.5	71.2	70.9	69.8	71.5	0.541	0.064
Carcass yield (%)							
Breasts	37.1	38.9	37.9	37.2	38.6	0.422	0.027
Legs	30.5	29.1	30.5	30.1	29.7	0.382	0.086
Wings	11.1	10.8	10.9	11.1	10.9	0.163	0.575
Back	20.4	20.7	20.1	20.7	20.1	0.316	0.498
Abdominal fat	0.92	0.92	0.87	0.91	0.91	0.122	0.999

Names of the groups described under Table 1.

**Table 3.** Effects of the dietary supplements on the water holding capacity and pH of the breast meat.

	Diets						
	Cont	+E	+C	+Se	+ECSe	SEM	*P*-value
WHC^1^	0.459	0.480	0.452	0.440	0.476	0.015	0.360
pH_24h_^2^	5.96	6.07	5.91	5.92	6.06	0.055	0.145
pH_48h_^3^	5.88	5.95	5.87	5.84	5.95	0.043	0.314

1 WHC = water holding capacity, expressed as M/T (M = area of the pressed meat film, T = wet area on the filter paper).

^2^pH_24h_ = pH measured 24 h post-mortem.

^3^pH_48h_ = pH measured 48 h post-mortem.

Names of the groups described under Table 1.

**Table 4.** Breast meat color parameters (L*, a*, b*; Minolta system) in broilers fed the experimental diets as affected by refrigerated storage.

	Diets						
Color parameters	Cont	+E	+C	+Se	+ECSe	SEM	*P*-value
L^*^_24h_	62.3^X^	61.4^X^	62.9^X^	61.7^X^	61.7^X^	0.959	0.838
L^*^_48h_	59.4^X,Y^	58.5^X,Y^	59.5^Y^	58.8^X,Y^	59.5^X,Y^	0.992	0.937
L^*^_72h_	57.9^Y,Z^	56.4^Y,Z^	58.1^Y,Z^	57.4^Y^	58.2^X,Y^	1.038	0.722
L^*^_132h_	55.3^Z^	54.0^Z^	55.6^Z^	56.0^Y^	55.8^Y^	0.925	0.590
*P*-value (time)	<.0001	<.0001	<.0001	0.0002	0.038		
a^*^_24h_	0.847	0.866	1.11	1.406	1.103	0.278	0.623
a^*^_48h_	1.24	1.37	1.542	1.78	1.45	0.259	0.658
a^*^_72h_	0.916	1.199	1.388	1.425	1.069	0.231	0.504
a^*^_132h_	1.26	1.42	1.72	1.52	1.06	0.229	0.340
*P*-value (time)	0.388	0.489	0.108	0.770	0.531		
b^*^_24h_	5.40^X^	4.90^X,Y^	6.23	5.79^X^	5.33	0.418	0.258
b^*^_48h_	5.34^X^	5.38^X^	6.03	5.73^X,Y^	5.40	0.417	0.723
b^*^_72h_	4.97^X,Y^	4.67^X,Y^	5.69	5.14^X,Y^	4.85	0.407	0.476
b^*^_132h_	4.03^Y^	3.48^Y^	4.85	3.99^Y^	4.16	0.393	0.220
*P*-value (time)	0.002	0.019	0.065	0.036	0.203		

X–Z Least squares means within a column without the same superscript differ significantly (*P* < 0.05).

L^*^ = Lightness; a^*^ = Redness; b^*^ = Yellowness, measured in different intervals post-mortem: 24 h, 48 h, 72 h, and 132 h.

Names of the groups described under Table 1.

The fatty acid profile of cooked fresh breast was not affected by the dietary supplements. Only in the case of C22:5 n-3, lower levels were observed in group +C (30%) compared to group +E (Table 5).

**Table 5.** Fatty acid profile (FA) of cooked fresh breast meat (g FA/100 g total FA).

	Diets						
Fatty acids^1^ (g/100 g FA)	Cont	+E	+C	+Se	+ECSe	SEM	*P*-value
C16:0	18.71	18.49	18.92	18.91	18.89	0.262	0.734
Ʃ C16:1^2^	2.22	2.49	3.05	2.80	2.71	0.204	0.096
C18:0	9.02	8.69	8.10	8.40	9.02	0.256	0.051
Ʃ C18:1^2^	25.3	24.1	25.9	25.7	26.1	0.540	0.071
C18:2 n-6	19.37	20.20	19.46	20.12	19.44	0.552	0.710
C18:3 n-3	12.34	12.38	13.06	13.01	12.19	0.675	0.831
C20:4 n-6	2.97	3.26	2.45	2.49	2.79	0.228	0.096
C20:5 n-3	1.66	1.70	1.45	1.29	1.46	0.112	0.110
C22:4 n-6	0.408	0.441	0.335	0.364	0.390	0.029	0.143
C22:5 n-3	2.32^a,b^	2.50^a^	1.75^b^	1.96^a,b^	2.22^a,b^	0.155	0.023
C22:6 n-3	0.896	0.933	0.701	0.700	0.930	0.083	0.033
SFA (Σ)	29.7	29.2	28.8	29.2	29.2	0.492	0.798
MUFA (Σ)	28.0	27.0	29.4	28.9	29.2	0.721	0.071
PUFA (Σ)	42.4	43.8	41.3	42.0	41.6	0.756	0.192
n-3 PUFA (Σ)	18.3	18.6	17.9	17.8	17.7	0.511	0.741
n-6 PUFA (Σ)	24.1	25.3	23.4	24.1	23.9	0.583	0.280
n-6/n-3	1.33	1.37	1.33	1.36	1.36	0.055	0.976

a,b Least squares means within a row without the same superscript differ significantly (*P* < 0.05).

1 Only the predominant fatty acids are presented, but the sums are calculated from all the fatty acids analysed: saturated fatty acids (SFA), monounsaturated fatty acids (MUFA) and polyunsaturated fatty acids (PUFA).

2 Sum of all isomers.

Names of the groups described under Table 1.

The MDA content of the breast meat was significantly affected by the inclusion of vitamin E (+E and +ECSe), as it decreased MDA content in all observed treatments compared to Cont (decrease ranged from 49.3% in +E in fresh meat to 65.9% in +E in cooked frozen stored meat). The cooking process increased the MDA content in breast meat approximately 3 to 5 times compared to non-treated breast meat. Dietary vitamin C and Se did not have any significant effect on the MDA content (Table 6).

**Table 6.** MDA content in fresh (Leskovec et al., 2018), frozen stored, cooked fresh, and cooked frozen stored breast muscle (nmol/100 g).

	Cont	+E	+C	+Se	+ECSe	SEM	*P*-value
Fresh	118.7^b^	60.2^a^	116.5^b^	92.1^a,b^	66.8^a^	10.88	0.002
Frozen stored	128.2	98.3	133.1	118.1	77.2	14.31	0.058
Cooked fresh	527.8^b^	210.4^a^	566.7^b^	621.8^b^	180.0^a^	60.84	<.0001
Cooked frozen stored	508.6^b^	177.9^a^	516.1^b^	531.2^b^	226.7^a^	57.92	0.0002
*P*-value for frozen storage and cooking							
Frozen storage	0.916	0.883	0.820	0.509	0.175		
Cooking	<.0001	<.0001	<.0001	<.0001	<.0001		
Frozen storage x Cooking	0.749	0.120	0.602	0.259	0.413		

a,b Least squares means within a row without the same superscript differ significantly (*P* < 0.05).

Names of the groups described under Table 1.

The tocopherols content of breast meat was improved only by the inclusion of vitamin E (+E and + ECSe). The dietary supplementation of α-tocopheryl acetate (vitamin E) increased the α-tocopherol concentration (from 143 to 252% in comparison to Cont) in breast meat. The frozen storage and the cooking process did not have any significant effect on the content of tocopherols (Table 7).

**Table 7.** Content of α- and γ-tocopherol in fresh (Leskovec et al., 2018), frozen, cooked fresh, and cooked frozen breast meat.

		Diets						
Breast muscle (µg/100 g)		Cont	+E	+C	+Se	+ECSe	SEM	*P*-value
Fresh	α-toc.	287.3^a^	773.3^b^	275.6^a^	278.7^a^	950.1^b^	42.1	<.0001
	γ-toc.	71.6	57.6	67.8	71.0	56.7	4.24	0.073
Frozen stored	α-toc.	304.1^a^	740.2^b^	285.1^a^	277.2^a^	992.6^b^	48.53	<.0001
	γ-toc.	70.5	57.5	69.7	67.1	60.8	3.51	0.060
Cooked fresh	α-toc.	280.9^a^	703.1^b^	247.4^a^	278.6^a^	989.6^b^	49.50	<.0001
	γ-toc.	65.3	55.0	61.6	63.6	57.3	3.26	0.176
Cooked frozen stored	α-toc.	288.9^a^	800.2^b^	283.7^a^	268.5^a^	1017.8^b^	54.42	<.0001
	γ-toc.	68.3	60.5	64.0	64.8	60.0	4.05	0.585
*P*-value for frozen storage and cooking, α-tocopherol								
Frozen storage		0.323	0.576	0.291	0.985	0.226		
Cooking		0.314	0.873	0.466	0.903	0.492		
Frozen storage x Cooking		0.583	0.408	0.508	0.997	0.535		
*P*-value for frozen storage and cooking, γ-tocopherol								
Frozen storage		0.782	0.363	0.693	0.798	0.240		
Cooking		0.127	0.996	0.012	0.296	0.982		
Frozen storage x Cooking		0.541	0.327	0.908	0.895	0.768		

a,b Least squares means within a row without the same superscript differ significantly (*P* < 0.05).

Names of the groups described under Table 1.

The ACW was not affected by any of the dietary supplemented groups. However, ACW was reduced after cooking (approximately by a quarter of the initial values in all the experimental groups) (Table 8).

**Table 8.** Antioxidant capacity of water soluble antioxidants (ACW) in fresh (Leskovec et al., 2018), frozen stored, cooked fresh and cooked frozen stored breast meat.

	Diets						
ACW (µmol ascorbic acid/100 g)	Cont	+E	+C	+Se	+ECSe	SEM	*P*-value
Fresh	22.6	23.1	25.8	23.1	24.6	1.66	0.647
Frozen stored	20.1	20.1	21.7	20.1	20.1	1.18	0.844
Cooked fresh	16.3	15.9	16.6	15.2	16.6	0.959	0.820
Cooked frozen stored	15.1	15.2	16.2	15.0	15.9	0.899	0.812
*P*-value for frozen storage and cooking, ACW							
Frozen storage	0.034	0.066	0.045	0.015	0.021		
Cooking	<.0001	<.0001	<.0001	<.0001	<.0001		
Frozen storage x cooking	0.457	0.291	0.073	0.030	0.092		

Names of the groups described under Table 1.

## DISCUSSION

Broilers meat, especially that enriched with n-3 PUFA, is highly susceptible to oxidative damage and consequently to quality deterioration (Panda and Cherian, 2014). Therefore, the supplementation with dietary antioxidants could improve broilers oxidative status and meat quality. Even though commercial recommendations for practical rearing conditions (Aviagen, 2014) already take into account higher needs for vitamin E than the basal requirements (NRC, 1994), some trials reported further benefits of a supranutritional inclusion of vitamin E in broilers fed diets rich in n-3 PUFA (Voljč et al., 2011). Furthermore, there are no recommendations for vitamin C and Se in cases of high dietary n-3 PUFA levels, although the benefits of their inclusion on the in vivo oxidative status and meat quality, especially under heat-induced oxidative stress, were reported (Estévez, 2015). Despite the fact that the antioxidant defense network is constituted by many biochemical reactions and pathways, only scarce data on the possible synergistic effects among antioxidants in broilers are available.

In the present study, none of the supplemented antioxidants had major effects on the productive parameters, except from vitamin E, which affected the feed intake negatively (Leskovec et al., 2018). Nevertheless, although differences in growth among groups did not seem relevant, in some cases the fatty acid profile, dressing percentage and meat quality could be affected, as the animal growth is allometric (Jones, 2004).

Carcass characteristics are important parameters in practice and even the smallest differences in the carcass dressing and carcass parts can lead to important economic changes. We did not obtain significant differences in the carcass dressing and shares of the carcass parts, except for the group +E (*P* = 0.055), which had a higher breast yield than Cont. That does not comply with Scheuermann et al. (2003), who showed that with higher carcass age and mass, a higher breast yield is expected. Since animals in groups +E and +ECSe had the numerically lowest weight at the end of the trial, it is not possible to separate the effect of the differences in growth or the direct effect of the dietary vitamin E inclusion. On the other hand, Choct and Naylor (2004) reported that the supplementation with vitamin E and Se did not have any effect on the performance, breast, thighs and drumsticks yields in broilers. Supranutritional vitamin C and Se in diets rich in n-3 PUFA did not have any effect on the carcass parts yield, which suggests that the stress was not intense or that the in vivo response against PUFA-induced stress was different than that of heat stress.

Dietary antioxidants can have a positive influence on meat quality, especially in the case of high dietary n-3 PUFA levels. In such circumstances, a supranutritional supplementation of antioxidants could inhibit the negative effects on the performance and partially alleviate the oxidative stress, and consequently also improve the meat quality (Estévez, 2015). Contrary to the expectations, we did not detect any effect of the examined supplements on pH, WHC, and color of the breast meat. This is not in agreement with trials reporting that supranutritional dietary levels of vitamin E could improve the resistance against some abnormalities such as pale, soft, and exudative meat (Petracci and Cavani, 2012), improve the cell integrity, retard the lipid and pigment oxidation, and consequently positively affect the WHC and discoloration (Jensen et al., 1998). On the other hand, Kim et al. (2010 and Ryu et al. (2005)) observed no effect of dietary vitamin E on the color of meat in broilers. Similarly to the results of the present study, Young et al. (2003) reported no effect of the dietary vitamin C on pH and WHC, as well as Skrivan et al. (2012) on the sensorial acceptability and breast and thigh meat color. Kim et al. (2010) and Ryu et al. (2005) observed no effect of dietary Se on the color of meat, which coincides with our results. However, Miezeliene et al. (2011) detected a decreased lightness (L^*^), and increased redness (a^*^) and yellowness (b*) as an effect of the dietary supplementation with Se. We did not observe any synergistic effect of vitamins E, C, and Se on the pH, WHC, and color of the meat, but their effect could be different in cases of a diet lower in vitamin E or higher in n-3 PUFA. The changes in the color of the meat linked to its maturation during storage were comparable to those observed in other trials in broilers (Petracci and Fletcher, 2002), suggesting that the n-3 PUFA inclusion did not affect the maturation in a severe way, and that the antioxidants did not change the post mortem processes.

In the present study, the fatty acid profile of the meat was comparable to studies performed in poultry fed linseed oil diets (Kanakri et al., 2017) with considerable amounts of PUFA (Gonzalez-Ortiz et al., 2013; Tomažin et al., 2014). Concomitantly to the fatty acid profile, the MDA concentrations in breast in our trial were comparable to those reported in other trials reporting increased levels in broiler meat enriched with n-3 PUFA (Basmacioglu et al., 2004; Smet et al., 2008; Tomažin et al., 2014). The lowest lipid peroxidation in meat, measured as MDA, was that of the tocopheryl acetate-supplemented groups (+E and +ECSe), as also reported in previous trials (El-Senousey et al., 2018; Leskovec et al., 2018; Smet et al., 2008). In the present study, the fact that vitamin C and Se did not have any effect on the content of MDA in meat is different than results by Skrivan et al. (2012), who stated that both antioxidants in similar concentrations to ours (280 and 560 mg vitamin C/kg and 0.3 mg Se/kg) leaded to lower levels of thiobarbituric acid-reactive substances when compared to a non-supplemented control. We did not detect any synergistic effect of the supranutritional supplementation, which is in contrast to the reported synergistic effects of vitamin E and Se (Habibian et al., 2016; Harsini et al., 2012) and of vitamin E and C (Jena et al., 2013) under heat stress conditions. Heat stress could cause a different in vivo response than dietary n-3 PUFA and consequently effects of dietary antioxidants. Moreover, the total capacity of in vivo antioxidants in our trial was probably sufficient to prevent the oxidative stress, and therefore no benefits were observed in presence of dietary vitamin C and Se. Thermal treatment (Castellini et al., 2002) and prolonged frozen storage (Surai and Dvorska, 2002) often increase the degree of lipid peroxidation in poultry meat, which was observed in the present study. In addition, the effects of vitamins E, C, and Se on the content of MDA did not differ between frozen stored and heat-treated meat. This may suggest that the frozen storage and cooking did not affect the meat in a way that supplemented antioxidants could have an influence on oxidation processes.

Vitamin C has the ability to spare vitamin E (Forman et al., 2014), and Se-dependent enzymes regenerate vitamin C (Pompella et al., 2003), which also affects the tocopherol content in meat. In the present study, we observed that the inclusion of α-tocopheryl acetate (+E and +ECSe) increased the concentration of α-tocopherol greatly. It also lowered the concentration of of γ-tocopherol in the breast meat, even though only numerically, as observed in plasma (Leskovec et al., 2018), and as similarly to other trials in poultry (Jensen et al., 1998). Vitamin C and Se did not have any effect on tocopherols, which complies with the studies by Grau et al. (2001) and Skrivan et al. (2012), who reported an absence of sparing effect of vitamin C and Se on the concentration of vitamin E in meat. On the other hand, Skrivan et al. (2008) found a positive correlation of dietary organic Se and vitamin E in broiler breast meat. Cooking and prolonged storage did not affect the tocopherols content, indicating no major losses.

Water-soluble antioxidants, with vitamin C as one of the most important, have an important protective role against oxidative stress. Therefore, the ACW of breast meat, parameter that includes vitamin C, was measured but interestingly no differences were detected among groups. Probably, vitamin C is metabolized and excreted quickly, and possibly replaced by other in vivo antioxidants, blurring the direct in vivo effects. The cooking process lowered the ACW for approximately 25% in comparison to the meat prior cooking, since water-soluble antioxidants are poorly resistant to the heat treatment (Leškova et al., 2006).

In the present study, the inclusion of linseed oil in order to increase the oxidative load was not translated to extensive changes in meat quality. This result could be different in the case of a higher dietary linseed oil level, or if oxidized oil was included. On the other hand, the level of linseed oil included was applicable to practical feeding conditions to achieve high n-3 PUFA meat content, allowing the use of a health claim regarding the fatty acid profile. Since the prices of the linseed oil or linseed meal are high and this affects the price of the feed greatly, amounts of linseed oil below 5% are normally used. Furthermore, there are many trials showing that a dietary linseed oil inclusion even at low dosages (5 to 6%) exerts oxidative stress in broilers (Eder et al., 2005; Tomažin et al., 2013). Another possible drawback of our study is the basal level of antioxidants, mainly vitamin E, which was adjusted to commercial recommendations (Aviagen, 2014) and not to minimum requirements. On the other hand, the aim of the study was to test antioxidants in levels applicable to practical rearing conditions, and not under minimum requirements, which could give a different response in the observed parameters. Moreover, it is difficult to set the antioxidants supplementation levels, as they depend on many factors, such as the environmental conditions, age of the animals, duration of the supplementation and genetics, among others. In trials investigating ways to protect poultry against oxidative stress induced by high dietary PUFA levels, up to 400 mg tocopherols/kg feed, 1,000 mg ascorbic acid/kg feed and up to 0.5 mg Se/kg feed were normally used, depending largely on the dietary levels of PUFA (Barroeta, 2007; Estevez, 2015).

In conclusion, it seems that the commercial recommendations of dietary antioxidant levels ensure the protection of broilers against oxidative stress under conditions of high dietary n-3 PUFA intake. However, in the case of vitamin E, a higher inclusion than that recommended commercially retarded the lipid peroxidation measured as breast meat MDA content. Vitamin E was the most efficient antioxidant against oxidative stress caused by diets high in n-3 PUFA, even during frozen storage and cooking. The dietary supranutritional supplementation with vitamin C and Se did not seem to have any major influence on the meat quality and sensory properties. However, similarly as for in vivo antioxidative parameters (Leskovec et al., 2018), we can conclude that the supranutritional levels of antioxidants used, alone or in combination, do not exert any harmful effect on the animal performance and quality of meat. Thus, they can be safely used as nutritive antioxidants even when combined in high concentrations.
